# The Stability and Electronic Structure of Cu(200)/AuCu(200) Interface: An Insight from First-Principle Calculation

**DOI:** 10.3390/ma15041506

**Published:** 2022-02-17

**Authors:** Zihao Li, Junli Feng, Zhangxi Wu, Mingjun Pang, Dong Liu, Wenchao Yang, Yongzhong Zhan

**Affiliations:** 1 School of Resources, Environment and Materials, Guangxi University, Nanning 530004, China; lizihao925108188@163.com (Z.L.); 1815301041@st.gxu.edu.cn (Z.W.); mjpang@163.com (M.P.); 2 Guangxi Key Laboratory of Processing for Non-Ferrous Metals and Featured Materials, MOE Key Laboratory of New Processing Technology for Non-Ferrous Metals and Materials, Nanning 530004, China; 3 The Testing and Technology Center for Industrial Products, Shenzhen Customs, Shenzhen 518067, China; fjlhhp@foxmail.com (J.F.); hlyld@163.com (D.L.); 4 Shenzhen Academy of Inspection and Quarantine, Shenzhen 518010, China

**Keywords:** first principle, Cu/AuCu interface, adhesion strength, interfacial energy, density of states

## Abstract

AuCu phase had a significant effect on the bonding strength of Au80Sn20 alloy and Cu substrate. The formation of the AuCu(200)/Cu(200) interface significantly improves the shear strength of solder joints. Therefore, it is particularly important to analyze the strengthening mechanism of the AuCu phase in the Cu matrix. The atomic structure, interfacial stability, and interfacial bonding properties of the Cu(200)/AuCu(200) interface were investigated using first-principle calculation. The layer spacing convergence results show that seven layers of Cu(200) surface and seven layers of AuCu(200) surface are enough thick to be chosen for the interface model. The calculation shows that the surface energies are 1.463 J/m^2^ and 1.081 J/m^2^ for AuCu(200) surface and Cu(200) surface, respectively. Four interface combinations of Top sit, Long bridge, Short bridge, and Hollow were investigated by considering four stacking methods of AuCu(200). It is shown that the interfacial configuration of the Long bridge is the most stable and favorable structure, which has the largest adhesion work, the smallest interfacial energy, and the smallest interfacial spacing. The density of states and electron difference density were calculated for the four interfacial configurations, and the results showed that the main bonding mode of the Long bridge interface is composed of both Cu-Cu covalent bonds and Au-Cu covalent bonds.

## 1. Introduction

Au80Sn20 alloy is widely used in power electronic devices and optoelectronic packages because of its high thermal conductivity, good fluidity, excellent fatigue resistance, corrosion resistance, creep resistance, high yield strength, and no flux soldering [1,2,3,4,5]. Meanwhile, copper is often used as the main heat dissipation material and substrate material for soldering due to its good electrical conductivity and excellent heat dissipation properties [6,7]. The soldering of Au80Sn20 alloy to Cu substrate becomes the most common solder joint in high-reliability optoelectronic packages [8].

The reliability of the solder joint depends on the nature of the solder itself but is also closely related to the intermetallic compounds (IMCs) formed in the interfacial reaction [9]. The experimental results demonstrate that IMCs are mainly composed of AuCu and (Au,Cu)_5_Sn on the Au80Sn20/Cu interface [10,11,12,13,14,15]. However, these types of IMCs greatly affect the long-term reliability of the solder joints attributed to their intrinsic brittleness. In addition, these interfacial intermetallic compounds continue to grow with increasing thickness as the aging process progresses, triggering a deterioration in the reliability of the solder joints [16]. In addition, it has been shown that the preferential orientation of these IMCs to the Cu substrate is important for the interface bonding of the solder joint [15].

The experimental results show that the AuCu and (Au,Cu)_5_Sn phases are firstly formed at the interface at the beginning of the reflow. Then, the AuCu phase will partly transform into the AuCu_3_ phase in the aging stage. The AuCu_3_ phase will cause a sharp drop in the stability of the solder joints [11,14]. At present, the generation of the AuCu_3_ phase can be avoided by improving the process. Therefore, to further improve the reliability of solder joints, researchers focused on the regulation of the AuCu and (Au,Cu)_5_Sn phases.

Liu et al. [13] found that the shear strength of solder joints was 67.5 MPa in the presence of only (Au,Cu)_5_Sn phase at the interface, and then Huang et al. [15] found that the strength of solder joints increased to 90 MPa when a nano-AuCu layer was formed at the interface. Liu et al. [13] studied the fracture behavior of solder joints, and the results showed that the fracture mainly occurred at the (Au,Cu)_5_Sn/Cu interface. Later, Huang et al. [15] also explored the influence of the nano-AuCu layer on the fracture behavior. The shear results show that when there is a nano-AuCu layer at the interface, the solder is mainly fractured in the interface of the copper matrix (contains copper matrix and AuCu layer) and the solder matrix. The location of the major tear is the copper matrix. The strong AuCu layers were partially destroyed, forming small cleavage surfaces. This indicates that the stability of the solder joint is obviously improved when the AuCu/Cu interface is formed. Unfortunately, experimental studies are still scarce on this interface, and the understanding of the bonding mechanism is still unclear between AuCu and Cu substrates. It is very difficult to analyze this interface only from experiments. Therefore, it is very important to resolve the interface in combination with other methods. Theoretical calculations provide a powerful contribution to the understanding of the interfacial bonding mechanism.

Therefore, it is necessary to study the Cu/AuCu interface structure theoretically. Huang et al. [15] used SEAD to determine the orientation relationship of the Cu substrate/AuCu interface as (200)Cu‖(200)AuCu and [001]Cu‖[001]AuCu.

In this paper, the Cu(200)/AuCu(200) interface is studied, and subsequently, the binding energy, interfacial energy, electronic structure, and bonding properties of the Cu(200)/AuCu(200) interface are calculated using a first-principle approach.

## 2. Calculation Details

All theoretical calculations were based on the first-principle method in density functional theory (DFT), which was implemented by using Cambridge Serial Total Energy Package (CASTEP) Codes [17,18]. Then, the ultrasoft pseudopotential, which is common to all, was chosen to consider ion–electron interactions [19]. For Cu and Au, the valence electrons considered are 3d^10^4s^1^ and 5d^10^6s^1^ in the pseudopotential, respectively. The Perdew–Burke–Ernzerhof (PBE) was used for the exchange-correlation functional within the generalized gradient approximation (GGA) formalism [20]. The Brillouin zone was sampled with a Monkhorst–Pack k-point grid [21]. To achieve the ground state and surface relaxation, the Broyden–Fletcher–Goldfarb–Shanno (BFGS) was employed for geometry optimization [22,23]. The convergence criterion for the total energy difference in geometric optimization was set to be less than 1 × 10^−5^ eV/atom.

We allowed all atoms to relax until the force on each atom is <0.03 eV/Å, the maximum stress should be <0.05 GPa, and the maximum ion displacement should be <0.001 Å. The cutoff energy was chosen to be 550 eV. K points of 12 × 12 × 12, 10 × 10 × 10, 10 × 14 × 1, 10 × 16 × 1, and 12 × 18 × 1 were used for the bulk Cu, bulk AuCu, Cu(200) surface, AuCu(200) surface, and Cu(200)/AuCu(200) interface, respectively, for full relaxation of the structure. The above choices of K points were proven to be reasonable after convergence tests. In addition, for the surface and interface of the plates, a 15 Å vacuum layer was chosen to isolate the free surfaces to ensure sufficient protection against their interaction. The total number of atoms on bulk Cu and Cu(200) surfaces are 4 and 14, respectively. For bulk AuCu and AuCu(200) surface, the total number of atoms was 4 and 14, respectively. Au atoms and Cu atoms were equally proportioned.

## 3. Results and Discussion 

### 3.1. Bulk Properties

**Figure 1:**
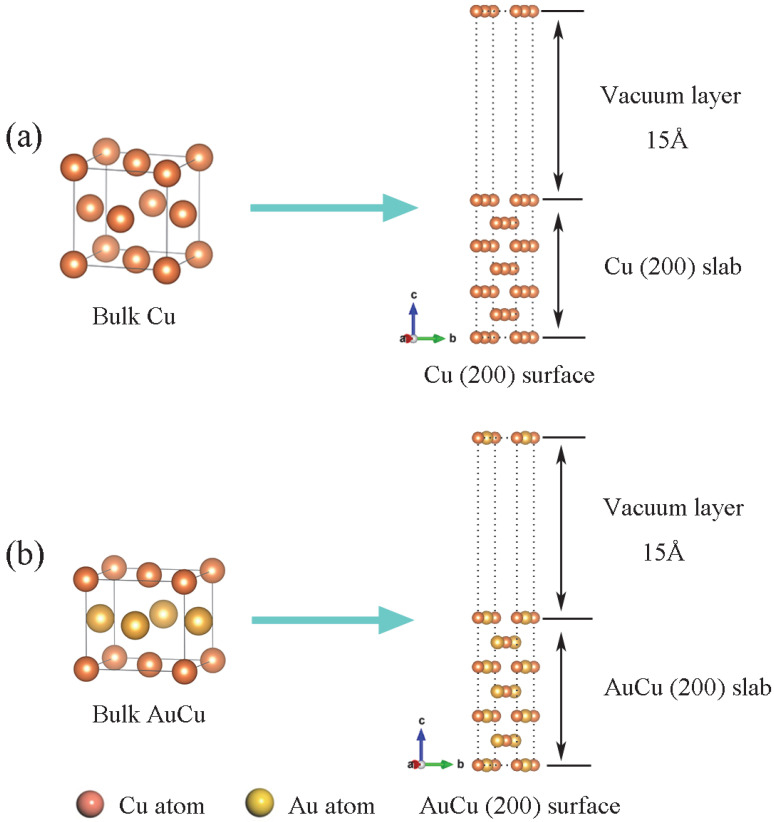
Schematic structures for Cu(200) surface (**a**) and AuCu(200) surface (**b**).

The lattice parameter of the bulk Cu optimized in this study is a = 3.63 Å, which is in good agreement with a previous computational value of 3.63 Å and an experimental value of 3.615 Å [24,25]. As shown in Figure 1b, Au atoms replace four face-centered positions of Cu atoms and form the L1_0_-AuCu cell. The lattice parameters of the L1_0_-AuCu intermetallic compound were calculated as a = 4.10 Å, c = 3.62 Å, which agree well with the calculated values of a = 4.09 Å, c = 3.73 Å and a = 4.0598 Å, c = 3.5204 Å, and the experimental values of a = 3.98 Å, c = 3.72 Å [26,27,28]. Hence, the calculation methodology and parameters are verified to generate accurate results. 

### 3.2. Surface Properties

$\left(\sqrt{2}\times 1\right)$ Cu(200) surface and $\left(\sqrt{2}\times 1\right)$ AuCu(200) surface were constructed by cutting optimized bulk Cu and AuCu. The edge lengths of Cu(200) surface is a_1_ = 5.134 Å, b_1_ = 3.63 Å, with an angle of 135°, and the edge lengths of AuCu(200) surface is a_2_ = 5.47 Å, b_2_ = 3.622 Å, with an angle of 132°. The lattice mismatches are less than 7%, and the angle mismatches are less than 3%. As shown in Figure 1, a vacuum layer was introduced on the surface of Cu(200) and AuCu(200) to eliminate the surface atomic interactions. In addition, for the slabs of surfaces and interfaces, a 15 Å vacuum was applied to separate the free surfaces to prevent their interactions.

In this study, the convergence of Cu(200) and AuCu(200) surfaces was tested by changing the interlayer spacing. It is defined as follows [29]:

(1) $$\Delta_{ij}\;=\frac{d_{ij}-d_{ij}^{0}}{d_{ij}^{0}}\times 100\%$$

where $d_{ij}^{0}$ denotes the spacing between adjacent atomic layers *i* and *j* after no relaxation, $d_{ij}$ denotes the spacing between adjacent atomic layers *i* and *j* after relaxation, and $\Delta_{ij}$ denotes the percentage change in the spacing of the atomic layers.

The results of layer spacing convergence for Cu(200) surfaces and AuCu(200) surfaces are shown in Table 1. For Cu(200) surfaces at Δ_23_ the layer spacing, variation is <1%. As a result, the seven atomic layers were sufficient to meet the bulk-like interior. For AuCu(200) surfaces, Au atoms and Cu atoms were, respectively used as reference points to calculate the layer spacing variation. As shown in Table 1, with the increase in atomic layers, Δ_23_ and Δ_34_ tend to converge, and the slab interiors meet bulk-like nature gradually. When the atomic layers are n ≥ 7, the slabs can reach bulk-like characteristics basically. Therefore, a seven-layer Cu(200) surface and a seven-layer AuCu(200) surface were adopted to construct their interface. 

The surface energy can be mutually corroborated by the layer spacing to ensure the preciseness of the calculation. Subsequently, the surface energies of the seven-layer Cu (200) surface and the seven-layer AuCu(200) surface were calculated. The calculation equations are as follows:

(2) $$\gamma_{surf}=\frac{E_{slab}-\left(\frac{N_{slab}}{N_{bulk}}\right)E_{bulk}}{2A}$$

where *E_slab_* and *E_bulk_* are total energies of the surface slab and the bulk unit cell, respectively. *N_slab_* and *N_bulk_* are numbers of atoms in the surface slab and the bulk unit cell, respectively. *A* is the surface area of a supercell.

It is calculated that the surface energy of the seven-layer Cu(200) surface is 1.463 J/m^2^, and this result is basically consistent with the earlier calculation of 1.46 J/m^2^ and experimental value of 1.79 J/m^2^ [24,30], which ensures the accuracy of the calculation. The surface energy of the seven-layer AuCu(200) surface is 1.081 J/m^2^. In summary, based on the calculation results of the layer spacing and surface energy, seven layers of Cu(200) surface and seven layers of AuCu(200) surface are sufficiently thick to be chosen for the interface model.

### 3.3. Interface Properties

#### 3.3.1. Atomic Structures

**Figure 2:**
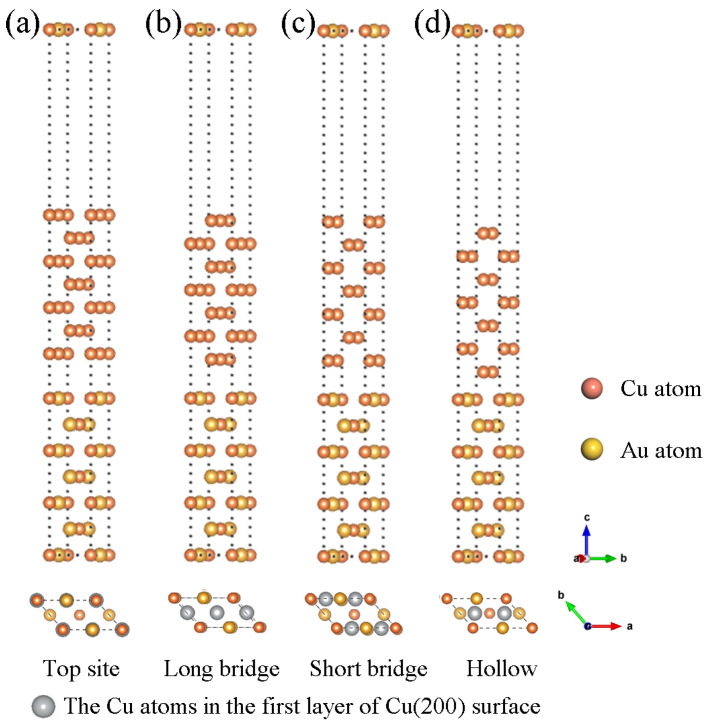
Side view of the four different Cu(200)/AuCu(200) interface model structure (up) and top view of the stack structure to which the interface model belongs (down): (**a**) Top site, (**b**) Long bridge, (**c**) Short bridge, (**d**) Hollow.

To construct a Cu(200)/AuCu(200) interface model, the AuCu(200) interface was used as the substrate, and the Cu(200) surface was then superimposed onto the AuCu(200) surface. Four stacking points may exist on the AuCu(200) surface, referred to as Top site, Long bridge, Short bridge, and Hollow. As shown in Figure 2a, for the Top site, the Cu atoms of the first layer on the Cu(200) surface are directly placed above the Cu atoms and Au atoms on the AuCu(200) surface. As shown in Figure 2b, for the Long bridge, the Cu atom at the interface on the Cu(200) side occupies the middle of the first Cu-Cu bond on the AuCu(200) side and the center of the Au-Au bond. As shown in Figure 2c, for the Short bridge, the Cu atom at the interface on the Cu(200) side occupies the center of the first Cu-Au bond on the AuCu(200) side. As shown in Figure 2d, for the Hollow site, the interfacial Cu atom on the Cu(200) side occupies the center of the diagonal of the first Cu-Au bond on the AuCu(200) side. Among these four interfacial structures, the continued choice of 15 Å vacuum layers was sufficient to eliminate the interaction between each interfacial layer.

#### 3.3.2. Adhesion Work and Interface Energy

The interfacial bonding strength can be qualitatively represented by adhesion work. The adhesion work was defined as the reversible work that separates the interface into two free surfaces. In this study, adhesion work was chosen to evaluate the bond strength of the Cu(200)/AuCu(200) interface, and the calculation formula is as follows [31]:

(3) $$W_{ad}=\frac{E_{Cu\left(200\right)}+E_{AuCu\left(200\right)}-E_{Cu\left(200\right)/AuCu\left(200\right)}}{A}$$

where *A* is the interfacial area, *E*_*Cu*(200)/*AuCu*(200)_ denotes the total energy of Cu(200)/AuCu(200) interface system, *E*_*Cu*(200)_ and *E*_*AuCu*(200)_ are the total energies of the relaxed and isolated Cu(200) and AuCu(200) slabs, respectively.

**Figure 3:**
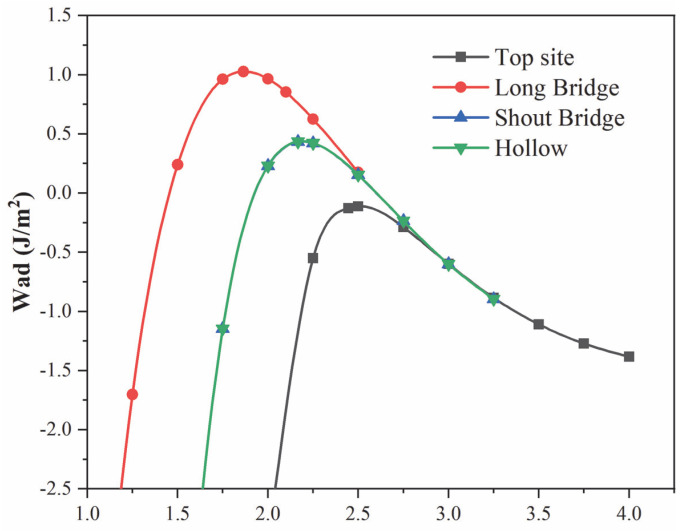
Universal binding energy relations for Cu(200)/AuCu(200) interface with four different stacking points.

*W_ad_* for Cu(200)/AuCu(200) interface can be obtained using a two-step approach that relates to unrelaxed geometries and relaxed geometries. Firstly, the total energies of different interfacial spacing configurations in Cu(200)/AuCu(200) interfaces were calculated without relaxation, the obtained data were fitted to the universal binding energy relationship (UBER), and the optimal *W_ad_* and d_0_ were derived [32]. Normally, the greater the adhesion work, the shorter the interfacial distance, and the better the interfacial bonding [33]. Figure 3 shows the UBER curves of four stacking orders of the Cu(200)/AuCu(200) interface. All four stacking sites show an ascending and then descending pattern. The Top site has the smallest *W_ad_* for adhesion and the largest interfacial spacing. It is worth noting that all adhesion work *W_ad_* of Top site is negative in the unrelaxed interface site model, which indicates that Top site is theoretically unstable in the unrelaxed interface stacking model. The UBER curves of the Short bridge and Hollow almost overlap, indicating that these two sites have the same *W_ad_* and interfacial spacing. They have the same interface stability. Then, compared with the other three stacking sites, the Long bridge site had the largest adhesion work and the smallest interfacial distance. This indicates that the Long bridge is the most thermodynamically stable surface. 

Then, according to the results of the UBER curve, the optimal interface spacing d_0_ was determined in the four unrelaxed interface models. Based on d_0_, the adhesion work W_ad_ and interface spacing d_1_ were obtained by fully relaxing the four interface models. The values of the adhesion work W_ad_ and the interfacial spacing d_0_ were calculated for the four model structures without and with full relaxation. As can be seen from Table 2, the adhesion work and interfacial spacing of the fully relaxed interface models have the same trend as those of the unrelaxed interface models, i.e., *W_ad_*(Lb) > *W_ad_*(Sb) = *W_ad_*(Hol) > *W_ad_*(Top), d_1_(Top) > d_1_(Sb) = d_1_(Hol) > d_1_(Lb); that is, the Long bridge is the most stable interface sequence among the four stacking sequences of Cu(200)/AuCu(200) interfaces.

In addition, interfacial energy is also an important indicator used to judge thermodynamic stability. It arises from changes in interfacial bonding and structural strain and is, therefore, difficult to measure experimentally. The interfacial energy $\gamma_{int}$ can be given as follows [33]:

(4) $$\gamma_{int}=\gamma_{sur}^{Cu\left(200\right)}+\gamma_{sur}^{AuCu\left(200\right)}-W_{ad}\;$$

where $\gamma_{sur}^{Cu\left(200\right)}$ and $\gamma_{sur}^{AuCu\left(200\right)}$ the surface energies of Cu(200) and AuCu(200), respectively.

Table 2 shows the interfacial energy of four different stacking models after total relaxation. In general, the smaller the interfacial energy, the more stable the interfacial structure and the better the wettability [34]. As can be seen from the table, compared with other interfaces, the Long bridge interface has the lowest interface energy of 1.083 J/m^2^. This also shows that the Long bridge model is the most thermodynamically stable structure. This is also consistent with our conclusion of adhesion work.

### 3.4. Electronic Structures and Bond Characteristics

To further understand the valence bond at the interface before and after the bonding of Cu(200) and AuCu(200) surfaces, the charge density differences of four interfaces (i.e., Top site, Long bridge, Short bridge, and Hollow) models were calculated in this study. The charge density difference is calculated as follows [35]:

(5) $$\Delta_{\rho }=\rho_{Cu\left(200\right)/AuCu\left(200\right)}-\rho_{Cu\left(200\right)}-\rho_{AuCu\left(200\right)}$$

where $\rho_{Cu\left(200\right)/AuCu\left(200\right)}$ is the total electron density of Cu(200)/AuCu(200) interface; $\rho_{Cu\left(200\right)}$ and $\rho_{AuCu\left(200\right)}$ are the electron densities of Cu(200) slab and AuCu(200) slab, respectively.

**Figure 4:**
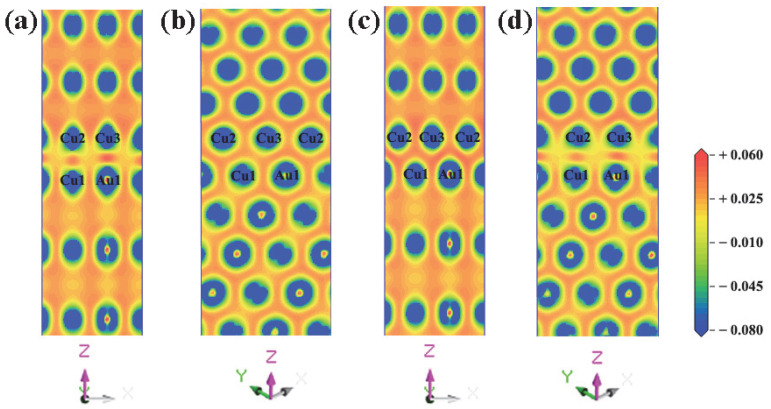
Charge density difference around the interface for four different Cu(200)/AuCu(200) interface models (unit: e/Å^3^): (**a**) Top site, (**b**) Long bridge, (**c**) Short bridge, and (**d**) Hollow.

The electron differential density maps of four models are drawn, as shown in Figure 4. Red represents charge accumulation and blue represents charge depletion. Short bridge and Hollow bridge models have similar adhesion work and interfacial energy, and Hollow models can reproduce the electronic differential density map of Short bridge models. Therefore, it is only necessary to compare the charge transfer between (a), (b), and (d). First, the three interface models were analyzed in terms of their similarities. For Top site and Hollow, the results show that there is a strong charge consumption near the Au atomic nucleus and the Cu atomic nucleus on the side of the AuCu(200) plate, and there is a strong charge accumulation in the bonding direction of the interface atoms, demonstrating that a strong Au-Cu covalent bond is formed between the interface atoms. The same phenomenon also occurs between Cu atom nuclei on the side of the AuCu(200) plate and Cu atom nuclei on the side of the Cu(200) plate, showing that strong Cu-Cu covalent bonds are formed between the interface atoms. For Top site and Hollow, these two interface models have similar charge transfer tendencies. The charge accumulation between Cu and Cu is weaker than that between Au and Cu in the two interface models, suggesting that the Au-Cu bond is stronger than the Cu-Cu bond. Then, the three interface models were compared in terms of differences. From the comparison of Figure 4a,d, it is found that the charge accumulation between Au and Cu is obviously stronger in the former than that in the latter. This indicates that the Au-Cu bond of the Top site model is stronger than the Au-Cu bond of the Hollow model. In addition, the charge accumulation between Cu and Cu is slightly stronger in the Top site model than that in the Hollow model. This manifests that the Cu-Cu bond of the Hollow model is slightly weaker than the Cu-Cu bond of the Top site model. Compared with the other three models, the Long bridge model leads to new discoveries. In the Long bridge model, the charge distribution is very uniform at the interface, and the charge accumulation of Cu-Cu and Cu-Au at the interface is very close to that of the inner atoms. Further analysis revealed that the charge transfer of atoms on the interface is very similar to the charge transfer of inner atoms. Consequently, there is no obvious interface tendency on the charge difference density (Figure 4b), but the bonding properties at the interface are the same as the inner atoms. This suggests that Au-Cu covalent bonds and Cu-Cu covalent bonds are formed in the Long bridge interface model, and these two bonds have similar strengths to the bonds of inner atoms. From comparing the bond strengths of the four interface models, it can be inferred that Au-Cu covalent bonds and Cu-Cu covalent bonds at the Long bridge interface are the strongest. Therefore, this is the underlying reason behind the superiority of the Long bridge interface model, compared with the other three models. 

**Figure 5:**
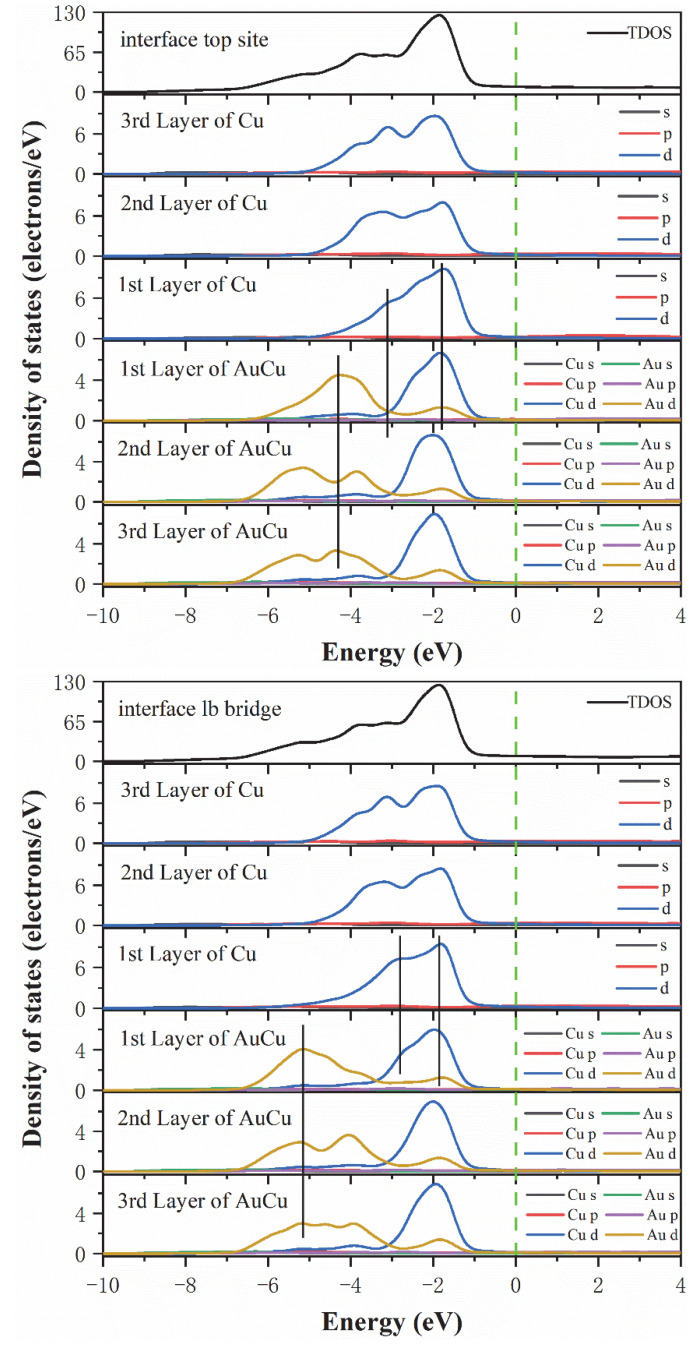

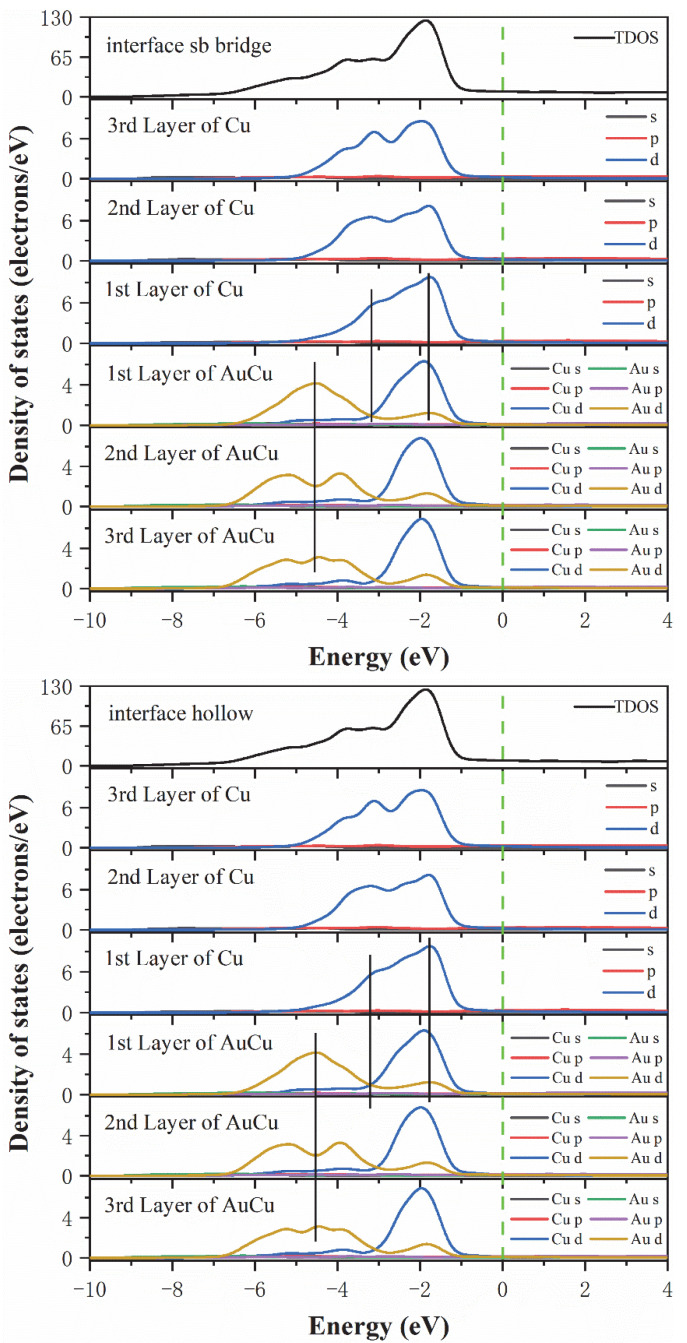
The total density of states and partial density of states of the Cu(200)/AuCu(200) interface. The vertical dashed line indicates the Fermi level and the vertical lines are drawn for guiding to the eye.

This analysis was followed by a further exploration of the bonding contributions between the Top site, Long bridge, Short bridge, and hollow interface atoms for each orbital, where the total density of states (DOSs) and partial density of states (PDOSs) were calculated and analyzed. As seen from Figure 5, the TDOSs of the four interface models were found to be very similar, indicating that these four interface models have similar electronic structures. Notably, the Short bridge interface model and the Hollow interface model have the same density of states, which also indicates that the Short bridge and Hollow models have the same bonding properties. This is consistent with the analysis results of electron differential density. Then, the optimal interface model was analyzed for the density of states. According to the Long bridge model, in the range of −3 eV to −1 eV, the Cu 3d-3d interaction produces obvious peaks, indicating that a strong Cu-Cu covalent bond is formed in the interface and the internal bulk. Different from the other three models, in the range of −3 eV to −2 eV, there is an overlapping peak at the interface of Cu atoms and the peak points are at the same level, which manifests that the 3d-3d orbital hybridization of Cu atoms in Long bridge model is more intense than the other three models. In the range of −7 eV to −1 eV, due to orbital hybridization of Au 5d and Cu 3d and the interaction between Au 5d-5d, strong Au-Cu covalent bonds, and Au-Au covalent bonds are formed in the interface and internal bulk. It is worth noting that after observing the peak positions of Au atoms at the interface and Au atoms in the inner layer, it is found that the peak points are at the same level for both the first and second Au atom layers. In the other three models, the peak point of the Au atom in the first layer is at the same level as the peak valley of the Au atom in the second layer. The reason for this discrepancy is not generated by the Au 5d-5d interaction but rather by the hybridization between Au5d and Cu3d. Therefore, the Long bridge model has a stronger hybridization than the other three models between Au 5d and Cu 3d. This shows that the bonding strength of the Long bridge interface is better than the other three models. In summary, the state density results show that the Long bridge interface model is superior to the other three models, which is consistent with the previous conclusion. 

To further clarify the bonding characteristics of the Cu(200)/AuCu(200) interface, the Mulliken population was analyzed. Mulliken population analysis is an important indicator to assess the bonding properties of an interface. Table 3 summarizes the Mulliken population results for the four different interface models. The population values of the four interface models are between 0.22 to 0.38, which demonstrates that strong Cu-Cu covalent bonds and Au-Cu covalent bonds are formed at the interface. It is worth noting that, in Table 3, there are two Cu1-Cu2 bonds and two Au1-Cu3 bonds each in the Long bridge interface model. For the Hollow interface model, the Cu1-Cu3 bond and the Au1-Cu3 bond are symmetrical to each other, and the Au1-Cu2 bond and the Cu1-Cu2 bond are also symmetrical to each other. Therefore, only Cu1-Cu2 bonds and Au1-Cu3 bonds can be displayed on the charge differential density map. From Table 3, the population value of the Long bridge interface model is the largest, indicating that the Cu-Cu and Au-Cu bonds in the Long bridge interface are stronger than the other three interface models. As regards the Short bridge and Hollow models, these two interface models have the same bonding type and Mulliken population. This also proves that they have the same bonding characteristics. It is worth noting that although the population values of the two interface models—Short bridge and Hollow—are smaller as Top sit, the combined results of the previous calculations show that the four bonds formed by the former contribute more than the two bonds formed by the latter. Thus, the Short bridge and Hollow interface models are better than the Top sit interface model. In summary, the Long bridge site is the best interface structure. These results are consistent with the analysis results of adhesion work, interface energy, the density of states, and electron differential density. 

## 4. Conclusions

The adhesion work, interfacial energy, electronic structure, and bonding properties of the Cu(200)/AuCu(200) interface were investigated using first-principle calculation. Four interfacial stacking models (Top site, Long bridge, Short bridge, and Hollow) were considered. The most stable interfacial structure was determined by evaluating the adhesion work and interfacial energy. Subsequently, the DOS, PDOS, and charge difference density of the four interfacial stacking models were calculated to gain insight into the nature of the interface. The following conclusions were obtained:

(1) According to the layer spacing determination, the surface energies of the seven-layer Cu(200) surface and AuCu(200) surface were calculated to be 1.463 J/m^2^ and 1.081 J/m^2^, respectively.
(2) The comparison of the adhesion work shows that the Long bridge has the largest adhesion work of 1.461 J/m^2^, which indicates that the interface model of the Long bridge is the most stable among the four interface stacking models. The order of the adhesion work of the four interface models is Long bridge > Short bridge = Hollow > Top site.
(3) The interfacial energies of four interface models were calculated, in which Long bridge had the smallest interfacial energy of 1.083 J/m^2^. The order of the interfacial energy of the four interface models is Long bridge < Short bridge = Hollow < Top site.
(4) Based on the results of electron difference density and density of states. For the Long bridge, it is known that the main bonding modes at the Cu(200)/AuCu(200) interfaces are Au-Cu covalent bonds and Cu-Cu covalent bonds.

## Figures and Tables

**Table 1:** The calculated surface atomic relaxations. Units are in % of the theoretical interlayer spacing of the corresponding surface.

Surface	Termination	Interlayer	Slab Thickness, n			
			3	5	7	9
Cu(200)	Cu	∆12	−2.37	−3.17	−2.81	−2.75
		∆23		0.42	0.79	0.72
		∆34			0.52	0.45
		∆45				0.05
AuCu(200)	Au	∆12	0.94	−1.07	−1.33	−1.04
		∆23		2.29	2.37	2.4
		∆34			0.31	−0.12
		∆45				0.28
	Cu	∆12	−4.3	−3.84	−3.76	−3.77
		∆23		−0.42	−0.73	−1.19
		∆34			0.41	0.71
		∆45				0.18

**Table 2:** The interfacial distance (d_0_, d_1_), adhesion work (*W_ad_*), and interfacial energy (*γ_int_*) of different Cu(200)/AuCu(200) interfaces under unrelaxed and relaxed conditions were investigated.

Stacking	UBRE		Fully Relaxed		
	d_0_ (Å)	*W_ad_* (J/m^2^)	d_1_ (Å)	*W_ad_* (J/m^2^)	*γ_int_* (J/m^2^)
Top site	2.445	−0.127	2.495	0.359	2.184
Long bridge	1.865	1.026	1.839	1.461	1.083
Short bridge	2.166	0.435	2.207	0.887	1.657
Hollow	2.166	0.437	2.207	0.886	1.657

**Table 3:** Results of Mulliken bond population analysis between nearest-neighbor atoms at four different Cu(200)/AuCu(200) interfaces.

Interface Type	Bond	Length (Å)	Population
Top site	Cu1-Cu2	2.495	0.25
	Au1-Cu3	2.530	0.33
Long bridge	Cu1-Cu2 (2)	2.581	0.33
	Au1-Cu3 (2)	2.646	0.38
Short bridge	Cu1-Cu3	2.575	0.22
	Cu1-Cu2	2.575	0.22
	Au1-Cu3	2.608	0.26
	Au1-Cu2	2.608	0.26
Hollow	Cu1-Cu2	2.575	0.22
	Cu1-Cu3	2.575	0.22
	Au1-Cu2	2.608	0.26
	Au1-Cu3	2.608	0.26

## Data Availability

The datasets generated during and analyzed during the current study are available from the corresponding author on reasonable request.
